# The prediction of virulence based on presence of virulence genes in *E. coli* may not always be accurate

**DOI:** 10.1186/s13099-015-0062-4

**Published:** 2015-06-19

**Authors:** Trudy M Wassenaar, Florian Gunzer

**Affiliations:** Molecular Microbiology and Genomics Consultants, Tannenstrasse 7, 55576 Zotzenheim, Germany; Institute of Medical Microbiology and Hygiene, TU Dresden, Dresden, Germany

**Keywords:** Virulence, *E. coli*, Whole Genome Sequencing, Pathogenicity

## Abstract

Now that microbial whole genome sequencing is in reach of many researchers, it is common to infer virulent properties of a given bacterial isolate based on the presence of virulence genes. However, this may lead to inaccurate presumptions of virulence. Using the findings of a recent publication (Da Silva Santos et al. Gut Pathog 7:2, 2015) where virulence was inferred from a genome sequence and subsequently confirmed by in vitro analysis, we present an alternative view on the case described in that publication. Our alternative view point, which is further substantiated by whole genome sequencing of probiotic *E. coli* strains, may contribute to a more balanced vision on the interactions between pathogens and host.

## Correspondence

Recently, Da Silva Santos and colleagues published a contribution to Gut Pathogens entitled ‘*Escherichia coli* from Crohn’s disease patient displays virulence features of enteroinvasive (EIEC), enterohemorrhagic (EHEC) and enteroaggregative (EAEC) pathotypes’ [1]. In this publication, an interesting *E. coli* strain was described, originating from a biopsy from a Crohn’s disease (CD) patient. The strain was analyzed by in vitro virulence assays and the analysis was completed by a whole-genome sequence.

These facts were described: the CD patient underwent terminal ileum resection. One particular *E. coli* strain was detected in all analyzed colonies, both from biopsies of the ileum and from stools. The bacteria were detected in an intestinal site where multiple erosions were observed. The isolated strain, named D92/09, was able to attach to and invade epithelial cells, although it was not a typical adherent and invasive *E. coli* (AIEC): it contained genetic markers that are more typical for diarrheagenic *E. coli*, namely those defining EHEC and EAEC pathotypes (specifically: presence of *eae*, *stx1*, *aggR*, *sat*, *espC*, *tsh* and *vat*). Further characterization confirmed adherent and invasive properties but only weak cytotoxic activity; cytotoxicity was stronger for internalized bacteria. The complete genome sequence identified 97% identity to an atypical EHEC strain that had been responsible for a large outbreak of enteropathic hemolytic uremic syndrome (HUS) in Germany [1].

The authors conclude that “(…) the bacteria were detected in an intestinal site where multiple erosions were observed. While this observation cannot imply a causal relationship, an eventual involvement of the bacteria cannot be ruled out. (…) [The] possession of a diversified virulence background like that of *E. coli* D92/09 would represent an adaptive advantage within the augmented bacterial population in this clinical condition [of CD]”.

Although this sounds altogether plausible, we would like to draw attention to a further fact, which sheds a different light on the findings. The patient is described as follows: “The studied *E. coli* was isolated in December 2009 from stools and an ileum biopsy of a 51-year-old woman who attended the Endoscopy unit of the University Hospital of the Botucatu Medical School/UNESP-SP, Brazil, for routine colonoscopy. The patient was diagnosed about 1 year earlier with small bowel CD complicated by an obstructive stenosis adjacent to the ileocecal valve and subsequently submitted to a terminal ileum and cecum resection. The patient had no clinical symptoms (…)”.

Thus, the patient had undergone the surgery (terminal ileum and cecum resection) to treat the obstructive stenosis, but the colonoscopy had been performed on a routine basis. If the biopsies had not been taken, this strain had probably not been detected. At the moment of colonoscopy, the patient was without symptoms; in other words, this invasive and weakly cytotoxic *E. coli* strain was present in biopsies, but it had not caused symptoms. This provides room for an alternative interpretation, namely that the immune system of this CD patient was quite capable to keep this presumably pathogenic *E. coli* in check, or, alternatively, that this strain was not as pathogenic as it appeared to be, as judged from its genetic content.

Whether a bacterial strain is pathogenic is not always easy to define, as has been pointed out before [2, 3]. The distinction between a pathogen and a commensal is not a sharp division, but rather a grey area, and this distinction has become even less clear with insights from whole-genome sequencing [4]. Colonization by commensals can protect against pathogens, though the same bacteria can cause disease in particular circumstances, as observed with, for example, the skin commensals *Staphylococcus aureus* and *Propionibacterium acnes* [5]. Likewise, colonization by probiotic yeasts is regarded as beneficial, but can, in particular cases, cause infections: fungemia caused by *Saccharomyces cerevisiae* and *S. boulardii* have both been described [6–8]. Whether the outcome of colonization is disease or a commensal relationship is defined by a combination of factors, of which the genetic properties of the bacteria are not the only ones: genetic and immunological factors of the host, as well as the presence of other microbiota are also contributing.

This discussion, which is not purely semantic, particularly applies to the species *E. coli*, which comprises commensal, beneficial (health promoting), as well as disease-causing strains. The genome of *E. coli* is highly variable [9], and undergoes frequent horizontal gene transfer: the genome of strain D92/09 bore evidence of recent gene acquisition by phages and other mobile elements, as the authors pointed out [1]. The notion that pathogenicity is a result of (virulence) gene acquisition has recently been countered with observations that pathogens often have reduced genomes, and virulence may result from loss of so-called antivirulence genes [10]. For instance, it has been demonstrated that in EAEC strains, expression of virulence genes is under control of regulator AggR, but in presence of antivirulence gene *aar*, a negative regulator, expression of *aggR* is down-regulated and virulence is reduced [11]. In view of the reported presence of *aggR* in strain D92/09, it would be interesting to see if *aar* is present as well, as this could explain the absence of symptoms in the patient.

Acknowledging that Da Silva Santos and colleagues have demonstrated (by PCR) presence of a number of well-established virulence genes in strain D92/09, we consider the presence of such genes not necessarily sufficient to assume a pathogenic phenotype. The probiotic *E. coli* strain Nissle1917 and the lesser known strain ABU 83972 are genetically very similar to uropathogenic *E. coli* strain CFT073: they share a large number of virulence genes and probably have a common ancestor [12]. This does not necessarily make these *E. coli* strains equally virulent: Nissle1917 (also known as EcN, trade name Mutaflor®) has GRAS status (generally recognized as safe) [13] and is classified as a Biosafety Level 1 organism by the Central Committee on Biological Safety (ZKBS) of the German Federal Office of Consumer Protection and Food Safety (BVL). Despite its classification and frequent use as a probiotic, including as treatment to maintain remission of ulcerative colitis [14], even *E. coli* Nissle 1917 is, in exceptional conditions, able to cause disease. For instance, it caused sepsis in a preterm newborn when administered at day 15 post-partum for treatment of a viral enteric infection [15].

We recently characterized the genome of six *E. coli* strains which together form the probiotic product Symbioflor 2®, each of which contained a number of virulence genes, as reported by the software MvirDB [16] and VFDB [17], computer programs specifically designed to identify virulence genes from bacterial genome sequences. These programs not only reported a range of virulence genes in the probiotic *E. coli* genomes, but also in the genome of *E. coli* K-12 MG1655, which we had included as a control [18]. Many of the identified virulence genes would only be functional when present in a complete genetic locus, which was often not the case. However, such incompleteness of virulence loci was not documented in the output of these programs, which considerably weakened the outcome of those analyses.

After correcting for completeness of loci, there remained a number of virulence genes that were clearly present in the six probiotic *E. coli* strains, including a complete *hly* locus encoding *E. coli* α-hemolysin in three of the strains [18]. Presence of this *hly* locus had been noted before, and the strains in question even express low levels of hemolysin [19]. Few data are available on presence of hemolysin in commensal *E. coli* strains, though anecdotal evidence suggests it is sometimes observed; older studies have reported that between 3.6 and 9.9% of enteric commensal isolates were haemolytic [20, 21]. A recent volunteer study demonstrated that in particular the weakly haemolytic strains present in the probiotic product colonized the human gut persistently [22]. Despite presence of *hly* and other virulence genes, the persistent colonization did not result in symptoms [22]. Although healthy volunteers were selected for this study, the probiotic product has been on the market for decades, and it is likely that IBD or CD patients have been amongst its many users. Nevertheless, safety reports collected over a period of six years covering over two million treatments sold reported very few adverse effects, and none that would indicate invasive or systemic infection [18].

In conclusion, presence of virulence genes in *E. coli* is difficult to predict, and even when identified, with in vitro evidence for (weak) expression, this is not always sufficient to predict a virulent phenotype for a given strain. Whether, in the case of the CD patient described by Da Silva Santos and coworkers, *E. coli* strain D92/09 should be considered pathogenic remains an open question in our view.
